# C-Reactive Protein to Albumin Ratio for Predicting Coronary Artery Lesions and Intravenous Immunoglobulin Resistance in Kawasaki Disease

**DOI:** 10.3389/fped.2020.607631

**Published:** 2020-11-25

**Authors:** Chih-Min Tsai, Hong-Ren Yu, Kuo-Shu Tang, Ying-Hsien Huang, Ho-Chang Kuo

**Affiliations:** ^1^Department of Pediatrics, Kaohsiung Change Gung Memorial Hospital, Kaohsiung, Taiwan; ^2^College of Medicine, Chang Gung University, Taoyuan, Taiwan; ^3^Kawasaki Disease Center, Kaohsiung Chang Gung Memorial Hospital, Kaohsiung, Taiwan; ^4^Department of Respiratory Therapy, Kaohsiung Chang Gung Memorial Hospital and Chang Gung University College of Medicine, Kaohsiung, Taiwan; ^5^Division of Pulmonary and Critical Care Medicine, Department of Internal Medicine, Kaohsiung Chang Gung Memorial Hospital and Chang Gung University College of Medicine, Kaohsiung, Taiwan

**Keywords:** C-reactive protein, albumin, Kawasaki disease (KD), coronary artery lesions, CRP to albumin ratio

## Abstract

**Background:** C-reactive protein (CRP) to albumin ratio (CRP/Alb) has emerged as a novel marker of inflammation, but few studies have evaluated the role of CRP/Alb ratio in Kawasaki disease (KD). Coronary artery lesions (CAL) in children with KD is a major acquired heart disease. We aimed to assess the association between CRP/Alb ratio and CAL formation in children with KD.

**Methods:** This retrospective study enrolled children diagnosed with KD and divided them into two groups, KD with CAL and KD without CAL. We compared the difference in gender, age, laboratory data, intravenous immunoglobulin (IVIG) resistance rate, and incidence of CAL between the two groups. Multivariable logistic regression analysis was used to assess the independent risk factors of CAL. We adopted receiver operating characteristic (ROC) curve analysis to determine the predictive ability of CRP/Alb ratio in predicting CAL.

**Results:** In total, 410 KD patients were reviewed, with 143 in the KD with CAL group and 267 in the KD without CAL group. KD children with CAL had a higher CRP/Alb ratio than those without CAL (3.14 ± 3.17 vs. 2.12 ± 2.04, *p* < 0.001). Multivariable logistic regression analysis demonstrated that male gender (OR = 3.222, *p* < 0.001), incomplete KD (OR = 1.968, *p* = 0.031), greater platelet count (OR = 1.004, *p* < 0.001), higher CRP (OR = 0.982, *p* = 0.048), and higher CRP/Alb ratio (OR = 1.994, *P* = 0.016) were all independent risk factors for predicting CAL. KD children with a high CRP/Alb ratio (≥2.94) had a higher incidence rate of CAL and IVIG resistance than those with a low CRP/Alb ratio (<2.94) (49.6 vs. 28.7%, *p* < 0.001 and 11.6 vs. 3.5%, *p* = 0.001, respectively).

**Conclusions:** This report is the first to show the role of CRP/Alb ratio in KD children. CRP/Alb ratio can serve as a novel predicting marker for CAL formation and IVIG resistance in KD.

## Introduction

Kawasaki disease (KD) is a form of acute febrile illness of unknown etiology that occurs primarily in children younger than 5 years old and is characterized by clinical laboratory and histopathological features of systemic vasculitis. In developed countries, KD is the most common cause of acquired heart disease in pediatric patients (1). The formation of coronary artery lesions (CAL), including coronary artery fistula formation, myocardial infarction, depressed myocardial contractility and heart failure, coronary artery dilatation, and coronary artery aneurysm (CAA) is the most severe complication or sequela in children with KD (2, 3). Although timely initiation of intravenous immunoglobulin (IVIG) treatment can reduce the incidence of CAA, it still occurs in 3–5% of KD patients (4). In our previous serial analysis of coronary artery dilation, about 1/3 of KD patients still developed CAL in the acute stage, and 4% of KD patients still had CAL or CAA formation for at least 1 year (5). Identifying those at risk of developing CAL is vital for preventing severe complication of KD with a more precise treatment protocol and adjunctive anti-inflammatory therapies (6–8). Several studies have attempted to predict risk factors for developing CAL in children with KD (9–15).

C-reactive protein (CRP) and albumin are commonly used parameters for measuring the activity of inflammatory conditions and are known as positive and negative acute phase reactants. The CRP to albumin (CRP/Alb) ratio has recently been considered a more useful indicator of sepsis than CRP or albumin alone (16). Studies have also shown that the CRP/Alb ratio can be used as a biomarker of the degree of activity in patients with autoimmune diseases (17, 18). One recent study has demonstrated that CRP/Alb ratio is a marker of disease activity in Takayasu arteritis (19). KD is also a form of systemic vasculitis that involves small to medium-sized vessels, particularly the coronary arteries. In the algorithm for diagnosis of incomplete KD recommended by American Heart Association (1), higher CRP level (≥30 mg/L) and lower serum albumin level (≤3 g/dL) are suggestive of diagnosis of incomplete KD. Previous studies have also proposed CRP and albumin as predictors of CAL in children with KD (10, 11, 13, 14), but the role of the CRP/Alb ratio in children with KD has not yet been evaluated. If CRP/Alb ratio is strongly associated with CAL, it would be especially useful because both CRP and albumin are not difficult to be determined and are frequently tested while evaluating patients with KD. Therefore, we aimed to assess the association between CRP/Alb ratio and CAL formation in children with KD in this study.

## Methods

The medical records of patients diagnosed with KD at Kaohsiung Chang Gung Memorial Hospital, Taiwan from January 2006 to December 2018 were retrospectively reviewed. This study was approved by the Chang Gung Medical Foundation's Institutional Review Board (IRB number: 201801163B0), which also approved the waiver of the informed consent form.

All KD children were diagnosed according to the American Heart Association guideline (1, 3). Initially, all subjects were treated with a single dose of IVIG (2 g/Kg) over a 12-h period. Aspirin (3–5 mg/kg/day) was given until regression of CAL was observed on two-dimensional (2D) echocardiography or all signs of inflammation had resolved.

The laboratory data used in this study were obtained within 1 day prior to initial IVIG administration and these parameters included complete blood cell count with differential (CBC/DC), CRP, albumin, aspartate aminotransferase (AST), and alanine aminotransferase (ALT). The dimensions of coronary artery of all KD patients were evaluated by 2D echocardiography before initial IVIG treatment. Within 8 weeks form the onset of the illness, at least two times of 2D echocardiography were performed to evaluate the change of coronary arteries. An abnormal coronary artery was defined as previously described in another study (20): if the lumen diameter was at least 3 mm in a child younger than 5 years old or at least 4 mm in a child ≥5 years old; the internal diameter of a segment was at least 1.5 times larger than that of an adjacent segment; or the morphology of the lumen was obviously abnormal. IVIG-resistant KD patients were defined as those who still have fever >48 h after administration of initial IVIG treatment and need to receive a second dose of IVIG or other anti-inflammatory regiments after the initial IVIG treatment had failed. Treatment failure was defined as recurrence of fever and one or more of the initial symptoms that led to the diagnosis of KD within 2–7 days of IVIG treatment (20). We divided all children with KD into two groups based on CAL formation: KD with CAL group and KD without CAL group.

### Statistical Analysis

All statistical analyses were carried out using IBM SPSS statistical software for Windows version 22.0 (Chicago, IL, USA). A *p*-value < 0.05 was considered statistically significant. Continuous variables were expressed as mean ± standard deviation, and categorical variables were reported as a percentage of patients. Categorical data were analyzed using the Chi-square test, and we utilized the Student's *t* test to compare the results of continuous variables. Statistical correlations were determined by the Pearson correlation test. To identify independent risk factors of CAL formation, we constructed univariable and multivariable logistic regression models and expressed the results as an odds ratio (OR) with a 95% confidence interval (CI). To assess the discriminatory capacity of the model and CRP/Alb ratio in predicting CAL, we performed the area under the receiver operating characteristic (ROC) curve analysis.

## Results

### Demographic Characteristics and Laboratory Tests

We reviewed a total of 504 children diagnosed with KD during the study period, of which 85 children had no albumin data, seven children had no CRP data, and two children had no CBC/DC results within 1 day prior to IVIG administration and were thus excluded. Ultimately, 410 children were enrolled in this study, with 60.0% being male (*n* = 247). Of these 410 cases, 143 children (34.79%) had CAL and 67 children were diagnosed as incomplete KD (16.3%). As shown in Table 1, the children in the KD with CAL group had a higher proportion of male gender and incomplete KD than children without CAL (75.5 vs. 51.7%, *p* < 0.001 and 22.4 vs. 13.1%, *p* = 0.016, respectively). The CRP/Alb ratio was significantly higher in KD children with CAL than KD children without CAL (3.10 ± 3.17 vs. 2.13 ± 2.04, *p* < 0.001). Other characteristics, such as admission days, white blood cell count, red blood cell count, hemoglobin, hematocrit, platelet count, percentage of neutrophil, lymphocyte, and basophil, CRP, and albumin level differed significantly in these two groups. As shown in Table 2, the CRP/Alb ratio is positively correlated with ESR, WBC, and neutrophil percentage, indicating that this ratio is associated with inflammation.

**Table 1:** Basic characteristics in Kawasaki disease patients with or without coronary artery lesions (CAL).

	**All KD patients**	**KD with CAL**	**KD without CAL**	***p*-value**
N	410	143	267	
Age	1.88 ± 1.59	1.87 ± 1.73	1.88 ± 1.51	0.924
Gender				<0.001^†^
Male	246 (60.0%)	108 (75.5%)	138 (51.7%)	
Female	164 (40.0%)	35 (24.5%)	129 (48.3%)	
KD type				0.016^*^
Complete	343 (83.7%)	111 (77.6%)	232 (86.9%)	
Incomplete	67 (16.3%)	32 (22.4%)	35 (13.1%)	
IVIG resistance	24 (5.9%)	10 (7.0%)	14 (5.2%)	0.472
Admission days	6.07 ± 3.38	7.10 ± 4.31	5.51 ± 2.59	<0.001^†^
ESR (mm/h)	53.24 ± 24.77	56.14 ± 24.77	51.78 ± 24.73	0.219
WBC (1,000/uL)	13.46 ± 4.73	14.20 ± 5.29	13.06 ± 4.36	0.020^*^
RBC (10^6^/uL)	4.27 ± 0.43	4.21 ± 0.47	4.31 ± 0.40	0.020^*^
Hemoglobin (g/dL)	11.09 ± 1.19	10.79 ± 1.37	11.26 ± 1.04	<0.001^†^
Hematocrit (%)	33.27 ± 3.33	32.59 ± 3.20	33.63 ± 3.34	0.002^*^
MCV (fL)	78.25 ± 5.25	77.82 ± 5.76	78.48 ± 4.95	0.224
MCH (pg/Cell)	26.08 ± 2.23	26.02 ± 2.29	26.12 ± 2.19	0.660
MCHC (gHb/dL)	26.08 ± 1.72	33.31 ± 0.95	33.25 ± 2.02	0.740
Platelet (1,000/uL)	351.35 ± 133.60	378.60 ± 158.30	336.76 ± 115.98	0.002^*^
Neutrophil (%)	59.27 ± 16.33	61.94 ± 16.84	57.84 ± 15.89	0.017^*^
Lymphocyte (%)	30.85 ± 14.93	28.31 ± 14.80	32.21 ± 14.84	0.012^*^
Monocyte (%)	5.94 ± 3.15	5.60 ± 2.76	6.1 ± 3.34	0.117
Eosinophil (%)	3.23 ± 3.34	3.39 ± 3.68	3.15 ± 3.16	0.492
Basophil (%)	0.20 ± 0.31	0.16 ± 0.28	0.22 ± 0.33	0.035^*^
AST (U/L)	63.98 ± 84.86	69.51 ± 94.73	60.97 ± 79.01	0.361
ALT (U/L)	78.10 ± 108.05	83.69 ±104.09	75.10 ± 110.20	0.441
CRP (mg/L)	84.15 ± 74.53	98.57 ± 84.64	76.43 ± 67.42	0.004^*^
Albumin (g/L)	37.14 ± 5.37	35.40 ± 6.32	38.07 ± 4.53	<0.001^†^
CRP/Alb ratio	2.48 ± 2.53	3.14 ± 3.17	2.12 ± 2.04	<0.001^†^

*KD, Kawasaki disease; IVIG, intravenous immunoglobulin; ESR, erythrocyte sedimentation rate; WBC, white blood cell; RBC, red blood cell; MCV, mean corpuscular volume; MCH, mean corpuscular hemoglobin; MCHC, mean corpuscular hemoglobin concentration; AST, aspartate transaminase; ALT, alanine transaminase; CRP, C-reactive protein; CRP/Alb, C-reactive protein/albumin*.

*Chi-square test and Student's t test were used to compare the categorical data and the results of continuous variables, respectively, between Kawasaki disease patients with or without coronary artery lesions*.

* *p < 0.05*.

† *p < 0.001*.

**Table 2:** Pearson's correlation between CRP/Alb ratio and other variables.

**Variables**	**ESR**	**WBC**	**RBC**	**Hemoglobin**	**Hematocrit**	**MCV**	**MCH**	**MCHC**
*r*	0.303	0.321	−0.325	−0.258	−0.270	0.048	0.064	0.081
*p*-value	<0.001	<0.001	<0.001	<0.001	<0.001	0.336	0.194	0.103
**Variables**	**Platelet**	**Neutrophil**	**Lymphocyte**	**Monocyte**	**Eosinophil**	**Basophil**	**AST**	**ALT**
*r*	−0.245	0.495	−0.479	−0.213	−0.069	−0.246	0.023	0.064
*p*-value	<0.001	<0.001	<0.001	<0.001	0.164	<0.001	0.649	0.199

*ESR, erythrocyte sedimentation rate; WBC, white blood cell; RBC, red blood cell; MCV, mean corpuscular volume; MCH, mean corpuscular hemoglobin; MCHC, mean corpuscular hemoglobin concentration; AST, aspartate transaminase; ALT, alanine transaminase; CRP/Alb, C-reactive protein/albumin*.

### Predictors for CAL Formation

**Figure 1:**
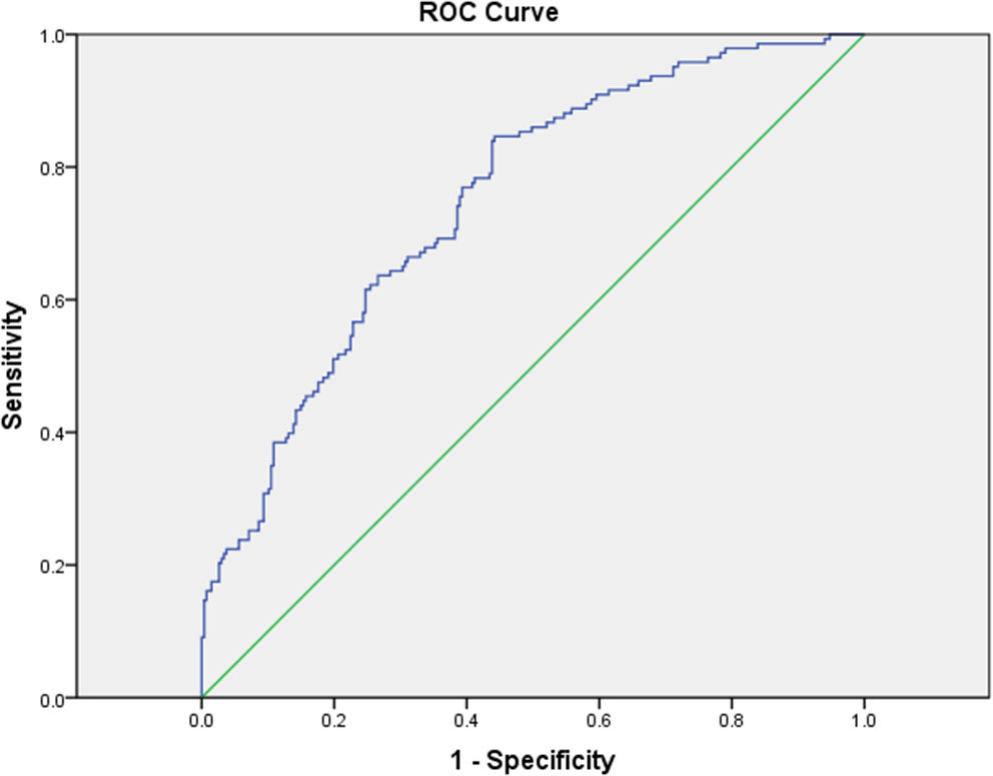
The receiver operating characteristic (ROC) curve analysis for the multivariable logistic regression model for the prediction of coronary artery lesions showed an area under the curve (AUC) = 0.748 with 95% CI = 0.700–0.797 (*P* < 0.001).

To evaluate the relative risk of each parameter, the parameters that demonstrated statistical differences were selected for univariable and multivariable logistic regression analysis. As shown in Table 3, the multivariable logistic regression analysis indicated that the following were independent risk factors of having CAL: male gender (OR = 3.222, 95% CI = 1.959–5.299, *p* < 0.001), incomplete KD (OR = 1.968, 1.064–3.639, *p* = 0.031), high platelet count (OR = 1.004, 95% CI = 1.002–1.006, *p* = 0.001), and high CRP/Alb ratio (OR = 1.994, 95% CI = 1.071–3.714, *p* = 0.030). The area under the ROC curve was 0.748 (95% CI = 0.700–0.797, *p* < 0.001) with sensitivity (84.6%) and specificity (55.4%) for predicting CAL formation of the multivariable logistic regression analysis (Figure 1). Gender had the highest OR in all variables, so we performed further multivariable logistic regression analysis in different genders. As shown in Table 4, the CRP/Alb ratio still had significant OR in both male and female patients in predicting CAL formation (OR = 1.184, 95% CI = 1.044–1.343, *p* = 0.008 for male and OR = 1.299, 95% CI = 1.069–1.579, *p* = 0.008 for female).

**Table 3:** Univariable and multivariable logistic regression analysis for predicting coronary artery lesions in children with Kawasaki disease.

	**Univariable LR**			**Multivariable LR**		
	**OR**	**95% CI**	***p*-value**	**OR**	**95% CI**	***p*-value**
Gender	2.884	1.838–4.526	<0.001^†^	3.222	1.959–5.299	<0.001^†^
Incomplete KD	1.911	1.125–3.247	0.017^*^	1.968	1.064–3.639	0.031^*^
WBC	1.052	1.008–1.098	0.021^*^	0.933	0.938–1.051	0.810
RBC	0.561	0.344–0.915	0.021^*^	1.497	0.719–3.116	0.281
Hemoglobin	0.704	0.583–0.850	<0.001^†^	0.772	0.516–1.156	0.209
Hematocrit	0.909	0.853–0.969	0.004^*^	1.029	0.883–1.200	0.713
Platelet	1.002	1.001–1.004	0.003^*^	1.004	1.002–1.006	0.001^†^
Neutrophil	1.016	1.003–1.029	0.016^*^	1.020	0.969–1.073	0.445
Lymphocyte	0.982	0.968–0.996	0.012^*^	1.004	0.949–1.062	0.896
Basophil	0.455	0.216–0.958	0.038^*^	0.535	0.233–1.229	0.141
CRP	1.004	1.001–1.007	0.005^*^	0.982	0.964–1.000	0.048^*^
Albumin	0.910	0.875–0.947	<0.001^†^	0.978	0.910–1.051	0.542
CRP/Alb ratio	1.169	1.076–1.270	<0.001^†^	1.994	1.071–3.714	0.030^*^

*LR, logistic regression; OR, odds ratio; CI, confidence interval; KD, Kawasaki disease; WBC, white blood cell; RBC, red blood cell; CRP, C-reactive protein; CRP/Alb, C-reactive protein/albumin*.

* *p < 0.05*.

† *p < 0.001*.

**Table 4:** Univariable and multivariable logistic regression analysis for predicting coronary artery lesions in children with Kawasaki disease based on different gender.

	**Univariable LR**			**Multivariable LR**		
	**OR**	**95% CI**	***p* value**	**OR**	**95% CI**	***p* value**
**MALE**						
Hemoglobin	0.748	0.599–0.934	0.010	0.848	0.574–1.252	0.407
Hematocrit	0.901	0.831–0.978	0.013	1.016	0.868–1.190	0.841
Platelet	1.002	1.000–1.004	0.024	1.003	1.000–1.005	0.020^*^
CRP/Alb ratio	1.143	1.026–1.273	0.015	1.184	1.044–1.343	0.008^*^
**FEMALE**						
WBC	1.128	1.044–1.220	0.002	1.036	0.944–1.138	0.453
Hemoglobin	0.592	0.403–0.869	0.008	0.667	0.436–1.021	0.062
Platelet	1.003	1.000–1.006	0.027	1.005	1.001–1.008	0.011^*^
Neutrophil	1.038	1.009–1.068	0.011	1.028	0.884–1.195	0.721
Lymphocyte	0.967	0.938–0.997	0.031	1.008	0.858–1.184	0.924
Eosinophil	0.810	0.660–0.995	0.045	0.843	0.645–1.101	0.211
CRP/Alb ratio	1.215	1.061–1.391	0.005	1.299	1.069–1.579	0.008^*^

*LR, logistic regression; OR, odds ratio; CI, confidence interval; WBC, white blood cell; CRP, C-reactive protein; CRP/Alb, C-reactive protein/albumin*.

* * p < 0.05*.

**Figure 2:**
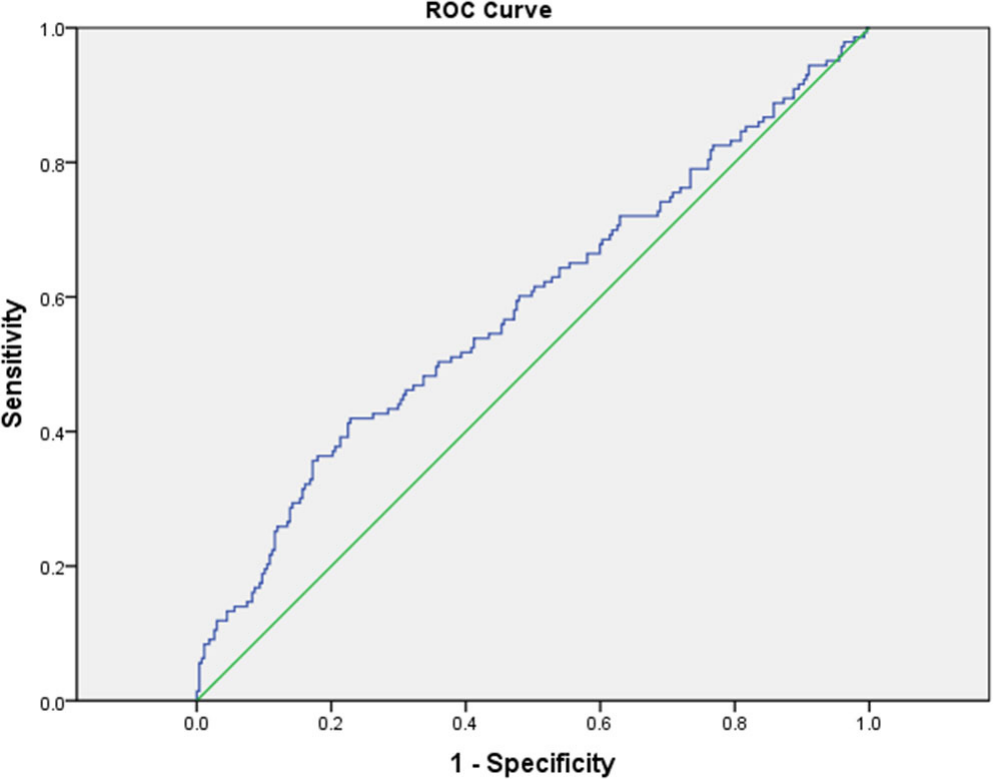
The receiver operating characteristic (ROC) curve analysis for CRP/Alb ratio for the prediction of coronary artery lesions showed an area under the curve (AUC) = 0.585 with 95% CI = 0.526–0.648 (*P* = 0.004).

Since CRP/Alb ratio, being an independent risk factor of CAL formation in children with KD, had the highest OR among laboratory variables, we performed ROC curve analysis to evaluate its ability to predict CAL formation. The area under the ROC curve was 0.585 (95% CI = 0.526–0.645, *p* = 0.004). With a cut-off level of 2.94 determined by Youden's index, the sensitivity and specificity in predicting CAL formation was 41.7 and 77.2%, respectively (Figure 2).

### Results of High CRP/Alb Ratio

**Figure 3:**
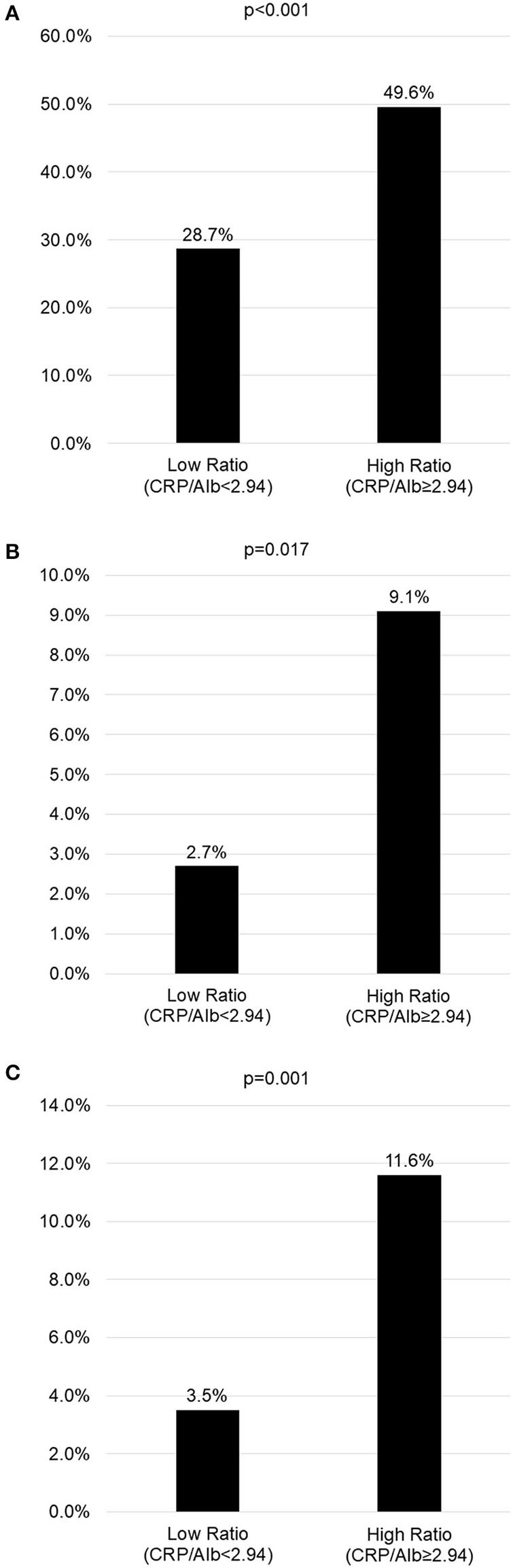
The incidence of coronary artery lesions, intravenous immunoglobulin resistance, and coronary artery aneurysms in children with high or low C-reactive protein to albumin (CRP/Alb) ratio (cut-off level of 2.94 determined by Youden index in the receiver operating characteristic (ROC) curve analysis). **(A)** Incidence of coronary artery lesions in children with high or low CRP/Alb ratio. **(B)** Incidence of coronary artery aneurysms in children with high or low CRP/Alb ratio. **(C)** Incidence of IVIG resistance in children with high or low CRP/Alb ratio.

As shown in Figure 3, we use the cut-off value determined by Youden's index to compare the incidence of CAL, CAA, and IVIG resistance in KD children with a high or low CRP/Alb ratio. KD children with a higher CRP/Alb ratio (≥2.94) had a higher incidence of CAL, CAA, and IVIG resistance. In such children, the odds of having CAL, CAA, and IVIG resistance were 1.73 times, 3.37 times, and 3.31 times greater, respectively, than children with a lower CRP/Alb ratio.

Among the 143 KD patients with CAL, 42 patients still had CAL 2 months after IVIG therapy, and 13 patients developed CAA. The incidence of CAA was about 3.17% (13/410) in our cohort. The mean CRP/Alb ratio of these 13 patients with CAA was 3.64 ± 3.22, which was higher than those without CAA (2.17 ± 2.09, *p* = 0.006).

## Discussion

To the best of our knowledge, this study is the first to investigate the role of CRP/Alb ratio in children with KD. The results of our study have indicated that (a) KD children with CAL had significantly higher CRP/Alb ratio values compared with KD children without CAL; (b) CRP/Alb ratio is an independent risk factor for developing CAL in children with KD; (c) KD children with a high CRP/Alb ratio had a higher incidence of CAL, CAA and IVIG resistance than those with a low ratio; and (d) CRP/Alb ratio helps predict CAL.

The most severe complication of KD is CAL, which has made KD the most common cause of acquired heart disease in children in developed countries (1). Therefore, the early identification of risk factors for CAL in KD children is crucial. In the current study, we found risk factors for CAL in children with KD, including male gender, incomplete KD, high platelet count, and high CRP/Alb ratio. Except for the CRP/Alb ratio, the other parameters have previously been reported to be risk factors for CAL formation in KD children (14, 15). In addition to help diagnose incomplete KD, high CRP level (10, 11, 13) and hypoalbuminemia (13, 14) have also been reported to be risk factors for CAL development in previous studies. Xie et al. reported the odds ratio for CRP > 14 mg/dL and albumin <30 g/L in predicting CAL in KD patients were 1.913 (95% CI = 1.113–3.290, *p* = 0.019) and 3.003 (95% CI = 1.596–5.651, *p* = 0.001), respectively (13). Another previous study reported hypoalbuminemia is associated with coronary artery abnormality with odds ratio of 1.07 per 1 g/dl decrease in albumin level (*p* < 0.001). Although we found KD children with CAL had higher CRP levels and lower albumin levels than children without CAL, our findings did not support these two parameters as being independent risk factors. This difference may be because we added their combination, CRP/Alb ratio, into the multivariable logistic regression model, and the regression coefficient of each parameter was thus adjusted (Table 3).

CRP is an acute-phase reactant protein that exhibits elevated expression in response to injury, infection, and inflammation (21). CRP levels also correlate with the degree of inflammation during the early course of illness in children (22). KD is characterized by systemic inflammation in all the medium-sized arteries, especially the coronary arteries and in multiple tissues and organs (23). Therefore, elevated CRP levels are commonly observed in children with KD. On the contrary, serum albumin is a negative acute-phase protein, and the intensity of inflammation response correlates with the degree of hypoalbuminemia (24). In KD vasculitis, increased microvascular permeability is an important event. Terai et al. reported that hypoalbuminemia in KD patients may be due to vascular leakage caused by the elevation of vascular endothelial growth factor (25). CRP and albumin levels diverge during the inflammation process in children with KD. Therefore, CRP/Alb ratio usage would offer a variable capable of combining the information of inflammation provided by CRP and albumin, i.e., a higher ratio indicates a higher inflammation status (26).

The CRP/Alb ratio has been used to assess residual inflammation at ICU discharge in septic patients (16) and mortality in critically ill patients (27). Recent studies have also demonstrated that CRP/Alb ratio correlated with the degree of activity in patients with inflammatory diseases. Yang et al. demonstrated a positive correlation between disease activity score and CRP/Alb ratio (*r* = 0.645, *P* < 0.001) in patients with rheumatoid arthritis (17). Qin et al. showed that CRP/Alb ratio had good discriminatory power for Crohn's disease activity [AUC = 0.747, 95% CI (0.649–0.845)] by ROC curve analysis (18). In one recent report, patients with Takayasu arteritis had a much higher CRP/Alb ratio than healthy controls (13.20 vs. 0.73, *p* < 0.001). The CRP/Alb ratio also was higher in patients with active Takayasu arteritis than patients in remission (11.26 vs. 1.34, *p* < 0.001) (19). In our study, we also found this ratio to be associated with CAL formation, which was related to the inflammation process in KD children. These findings all support the usefulness of CRP/Alb ratio in evaluating inflammatory disease.

In addition to patients' characteristics, such as gender and laboratory tests, different manufacturing processes of IVIG may also affect the treatment efficacy in children with KD (28–30). Tsai et al. has showed that patients receiving IVIG prepared with beta-propiolactone was associated with higher rates of IVIG-resistance (13%, *p* = 0.001), coronary artery abnormality (10%, *p* = 0.01) at convalescence, and giant aneurysm (3%, *p* = 0.008) (28). A nationwide cohort study including 3830 KD children also demonstrated beta-propiolactonation of IVIG had a relative risk of 1.45 (95% CI = 1.08–1.94) of IVIG-resistance and acidification of IVIG was associated with a higher acute coronary aneurysm with a relative risk of 1.49 (95% CI = 1.17–1.90) (29). Another previous study also found that IVIG preparations with lower IgA content is associated with improved coronary artery outcomes (30). Although we found CRP/Alb ratio was associated with CAL formation, our data lacked the manufacturing processes of IVIG we used. Further studies are needed to evaluate the association among patients' characteristics, manufacturing process of IVIG, IVIG resistance and CAL formation in children with KD.

Several risk scores (11, 12, 31), which were based on Asian population, have been established to predict IVIG resistance and CAL in children with KD. However, some previous studies failed to demonstrate a good discrimination of IVIG resistance and CAL in non-Japanese children while applying these risk scores (32, 33). One recent study also demonstrated that these scores have poor predictive ability in a Caucasian cohort (34). Our current study showed CRP/Alb ratio is a novel independent risk factor for CAL in Chinese. Would this finding also being found in other races and help improve the predictive ability of previous risk scores? Further studies involving completely new data from other races and institutions are warranted to assess the consistence of association between CRP/Alb ratio and children with KD. Additional studies are also needed to investigate the superiority of a high CRP/Alb ratio to the other reported risk factors capable of predicting CAL in KD children.

## Conclusion

A high CRP/Alb ratio is a risk factor for CAL and can thus serve as a novel marker for predicting CAL and IVIG resistance in children with KD.

## Data Availability Statement

The raw data supporting the conclusions of this article will be made available by the authors, without undue reservation.

## Ethics Statement

This study was approved by the Chang Gung Medical Foundation's Institutional Review Board (IRB number: 201801163B0), which also approved the waiver of the informed consent form. Written informed consent from the participants' legal guardian was not required to participate in this study in accordance with the national legislation and the institutional requirements.

## Author Contributions

Y-HH and H-CK conceptualized and design the study. C-MT and H-RY wrote the first draft of the manuscript. C-MT and K-ST collected and organized the database. All authors critically reviewed and revised the manuscript, and agreed to the published version of the manuscript. All authors contributed to the article and approved the submitted version.

## Conflict of Interest

The authors declare that the research was conducted in the absence of any commercial or financial relationships that could be construed as a potential conflict of interest.

## References

[1] McCrindle BW Rowley AH Newburger JW Burns JC Bolger AF Gewitz M. Diagnosis, treatment, and long-term management of Kawasaki disease: a scientific statement for health professionals from the American Heart Association. Circulation. (2017) 135:e927–99. 10.1161/CIR.0000000000000484 28356445

[2] Liang CD Kuo HC Yang KD Wang CL Ko SF. Coronary artery fistula associated with Kawasaki disease. Am Heart J. (2009) 157:584–8. 10.1016/j.ahj.2008.11.020 19249434

[3] Newburger JW Takahashi M Gerber MA Gewitz MH Tani LY Burns JC. Diagnosis, treatment, and long-term management of Kawasaki disease: a statement for health professionals from the Committee on Rheumatic Fever, Endocarditis and Kawasaki Disease, Council on Cardiovascular Disease in the Young, American Heart Association. Circulation. (2004) 110:2747–71. 10.1161/01.CIR.0000145143.19711.78 15505111

[4] Newburger JW Takahashi M Beiser AS Burns JC Bastian J Chung KJ. A single intravenous infusion of gamma globulin as compared with four infusions in the treatment of acute Kawasaki syndrome. N Engl J Med. (1991) 324:1633–9. 10.1056/NEJM199106063242305 1709446

[5] Kuo HC Yu HR Juo SH Yang KD Wang YS Liang CD. CASP3 gene single-nucleotide polymorphism (rs72689236) and Kawasaki disease in Taiwanese children. J Hum Genet. (2011) 56:161–5. 10.1038/jhg.2010.154 21160486

[6] Kobayashi T Saji T Otani T Takeuchi K Nakamura T Arakawa H. Efficacy of immunoglobulin plus prednisolone for prevention of coronary artery abnormalities in severe Kawasaki disease (RAISE study): a randomised, open-label, blinded-endpoints trial. Lancet. (2012) 379:1613–20. 10.1016/S0140-6736(11)61930-2 22405251

[7] Salgado AP Ashouri N Berry EK Sun X Jain S Burns JC. High risk of coronary artery aneurysms in infants younger than 6 months of age with Kawasaki disease. J Pediatr. (2017) 185:112–6.e1. 10.1016/j.jpeds.2017.03.025 28408126 PMC5529235

[8] Kuo HC Preventing coronary artery lesions in Kawasaki disease. Biomed J. (2017) 40:141–6. 10.1016/j.bj.2017.04.002 28651735 PMC6136281

[9] Beiser AS Takahashi M Baker AL Sundel RP Newburger JW. A predictive instrument for coronary artery aneurysms in Kawasaki disease. US Multicenter Kawasaki Disease Study Group. Am J Cardiol. (1998) 81:1116–20. 10.1016/S0002-9149(98)00116-7 9605052

[10] Mori M Imagawa T Yasui K Kanaya A Yokota S. Predictors of coronary artery lesions after intravenous gamma-globulin treatment in Kawasaki disease. J Pediatr. (2000) 137:177–80. 10.1067/mpd.2000.107890 10931408

[11] Kobayashi T Inoue Y Takeuchi K Okada Y Tamura K Tomomasa T. Prediction of intravenous immunoglobulin unresponsiveness in patients with Kawasaki disease. Circulation. (2006) 113:2606–12. 10.1161/CIRCULATIONAHA.105.592865 16735679

[12] Egami K Muta H Ishii M Suda K Sugahara Y Iemura M. Prediction of resistance to intravenous immunoglobulin treatment in patients with Kawasaki disease. J Pediatr. (2006) 149:237–40. 10.1016/j.jpeds.2006.03.050 16887442

[13] Xie T Wang Y Fu S Wang W Xie C Zhang Y. Predictors for intravenous immunoglobulin resistance and coronary artery lesions in Kawasaki disease. Pediatr Rheumatol. (2017) 15:17. 10.1186/s12969-017-0149-1 28320400 PMC5359815

[14] Sabharwal T Manlhiot C Benseler SM Tyrrell PN Chahal N Yeung RS. Comparison of factors associated with coronary artery dilation only versus coronary artery aneurysms in patients with Kawasaki disease. Am J Cardiol. (2009) 104:1743–7. 10.1016/j.amjcard.2009.07.062 19962487

[15] Giannouli G Tzoumaka-Bakoula C Kopsidas I Papadogeorgou P Chrousos GP Michos A. Epidemiology and risk factors for coronary artery abnormalities in children with complete and incomplete Kawasaki disease during a 10-year period. Pediatr Cardiol. (2013) 34:1476–81. 10.1007/s00246-013-0673-9 23463134

[16] Ranzani OT Zampieri FG Forte DN Azevedo LC Park M. C-reactive protein/albumin ratio predicts 90-day mortality of septic patients. PLoS ONE. (2013) 8:e59321. 10.1371/journal.pone.0059321 23555017 PMC3595283

[17] Yang WM Zhang WH Ying HQ Xu YM Zhang J Min QH. Two new inflammatory markers associated with disease activity score-28 in patients with rheumatoid arthritis: albumin to fibrinogen ratio and C-reactive protein to albumin ratio. Int Immunopharmacol. (2018) 62:293–8. 10.1016/j.intimp.2018.07.007 30048859

[18] Qin G Tu J Liu L Luo L Wu J Tao L. Serum albumin and C-reactive protein/albumin ratio are useful biomarkers of Crohn's disease activity. Med Sci Monit. (2016) 22:4393–400. 10.12659/MSM.897460 27848931 PMC12574461

[19] Seringec Akkececi N Yildirim Cetin G Gogebakan H Acipayam C. The C-reactive protein/albumin ratio and complete blood count parameters as indicators of disease activity in patients with Takayasu arteritis. Med Sci Monit. (2019) 25:1401–9. 10.12659/MSM.912495 30792377 PMC6396438

[20] Kuo HC Liang CD Wang CL Yu HR Hwang KP Yang KD. Serum albumin level predicts initial intravenous immunoglobulin treatment failure in Kawasaki disease. Acta Paediatr. (2010) 99:1578–83. 10.1111/j.1651-2227.2010.01875.x 20491705

[21] Sproston NR Ashworth JJ. Role of C-reactive protein at sites of inflammation and infection. Front Immunol. (2018) 9:754. 10.3389/fimmu.2018.00754 29706967 PMC5908901

[22] Rey C Los Arcos M Concha A Medina A Prieto S Martinez P. Procalcitonin and C-reactive protein as markers of systemic inflammatory response syndrome severity in critically ill children. Intens Care Med. (2007) 33:477–84. 10.1007/s00134-006-0509-7 17260130

[23] Amano S Hazama F Kubagawa H Tasaka K Haebara H Hamashima Y. General pathology of Kawasaki disease. On the morphological alterations corresponding to the clinical manifestations. Acta Pathol Jpn. (1980) 30:681–94. 10.1111/j.1440-1827.1980.tb00966.x 7446109

[24] Al-Subaie N Reynolds T Myers A Sunderland R Rhodes A Grounds RM. C-reactive protein as a predictor of outcome after discharge from the intensive care: a prospective observational study. Br J Anaesth. (2010) 105:318–25. 10.1093/bja/aeq171 20630889

[25] Terai M Honda T Yasukawa K Higashi K Hamada H Kohno Y. Prognostic impact of vascular leakage in acute Kawasaki disease. Circulation. (2003) 108:325–30. 10.1161/01.CIR.0000079166.93475.5F 12835221

[26] Fairclough E Cairns E Hamilton J Kelly C. Evaluation of a modified early warning system for acute medical admissions and comparison with C-reactive protein/albumin ratio as a predictor of patient outcome. Clin Med. (2009) 9:30–3. 10.7861/clinmedicine.9-1-30 19271597 PMC5922628

[27] Oh TK Song IA Lee JH. Clinical usefulness of C-reactive protein to albumin ratio in predicting 30-day mortality in critically ill patients: A retrospective analysis. Sci Rep. (2018) 8:14977. 10.1038/s41598-018-33361-7 30297724 PMC6175848

[28] Tsai MH Huang YC Yen MH Li CC Chiu CH Lin PY. Clinical responses of patients with Kawasaki disease to different brands of intravenous immunoglobulin. J Pediatr. (2006) 148:38–43. 10.1016/j.jpeds.2005.08.024 16423595

[29] Lin MC Fu YC Jan SL Lai MS. Comparative effectiveness of intravenous immunoglobulin for children with Kawasaki disease: a nationwide cohort study. PLoS ONE. (2013) 8:e63399. 10.1371/journal.pone.0063399 23650564 PMC3641142

[30] Manlhiot C Yeung RS Chahal N McCrindle BW. Intravenous immunoglobulin preparation type: association with outcomes for patients with acute Kawasaki disease. Pediatr Allergy Immunol. (2010) 21:515–21. 10.1111/j.1399-3038.2010.00987.x 20546528

[31] Lin MT Chang CH Sun LC Liu HM Chang HW Chen CA. Risk factors and derived formosa score for intravenous immunoglobulin unresponsiveness in Taiwanese children with Kawasaki disease. J Formos Med Assoc. (2016) 115:350–5. 10.1016/j.jfma.2015.03.012 25910931

[32] Davies S Sutton N Blackstock S Gormley S Hoggart CJ Levin M. Predicting IVIG resistance in UK Kawasaki disease. Arch Dis Child. (2015) 100:366–8. 10.1136/archdischild-2014-307397 25670405

[33] Sanchez-Manubens J Anton J Bou R Iglesias E Calzada-Hernandez J Borlan S. Role of the Egami score to predict immunoglobulin resistance in Kawasaki disease among a Western Mediterranean population. Rheumatol Int. (2016) 36:905–10. 10.1007/s00296-016-3499-y 27215220

[34] Fabi M Andreozzi L Corinaldesi E Bodnar T Lami F Cicero C. Inability of Asian risk scoring systems to predict intravenous immunoglobulin resistance and coronary lesions in Kawasaki disease in an Italian cohort. Eur J Pediatr. (2019) 178:315–22. 10.1007/s00431-018-3297-5 30499051

